# Mice with humanized-lungs and immune system - an idealized model for COVID-19 and other respiratory illness

**DOI:** 10.1080/21505594.2020.1763637

**Published:** 2020-05-20

**Authors:** Sujit Pujhari, Jason L Rasgon

**Affiliations:** Department of Entomology, The Center for Infectious Disease Dynamics, and the Huck Institutes of the Life Sciences

**Keywords:** SARS-CoV-2, respiratory infectious diseases, pluripotent stem cell, animal model

## Abstract

Lack of an appropriate animal model to study severe acute respiratory syndrome coronavirus 2 (SARS-CoV-2), the etiological agent responsible for COVID-19 pandemic disease, represents a significant hurdle in the process of understanding disease biology and evaluating therapeutic and preventive candidates. It is time for public health agencies to revisit regulation on transplantation of human pluripotent stem cells for the possibility of the development of a humanized mice model with a humanized lung.

Researchers around the globe are desperately searching for an animal model for evaluating potential therapeutics and vaccine candidates for severe acute respiratory syndrome coronavirus 2 (SARS-CoV-2), the etiological agent responsible for COVID-19, a respiratory illness currently causing a global pandemic. After crossing the species barrier from an as-yet-unidentified source, SARS-CoV-2 has established itself and is efficiently spreading among human populations at an alarming rate. With multiple pandemic epicenters across the globe, there are more than 1,979,000 confirmed human cases as of 14 April 2020[1]. The major complications of COVID-2019, and the main drivers for registered mortality are acute respiratory failure, high blood pressure and cardiovascular problems which are mostly (but not exclusively) restricted to patients over 50 and patients with underlying conditions.

**Figure 1.**
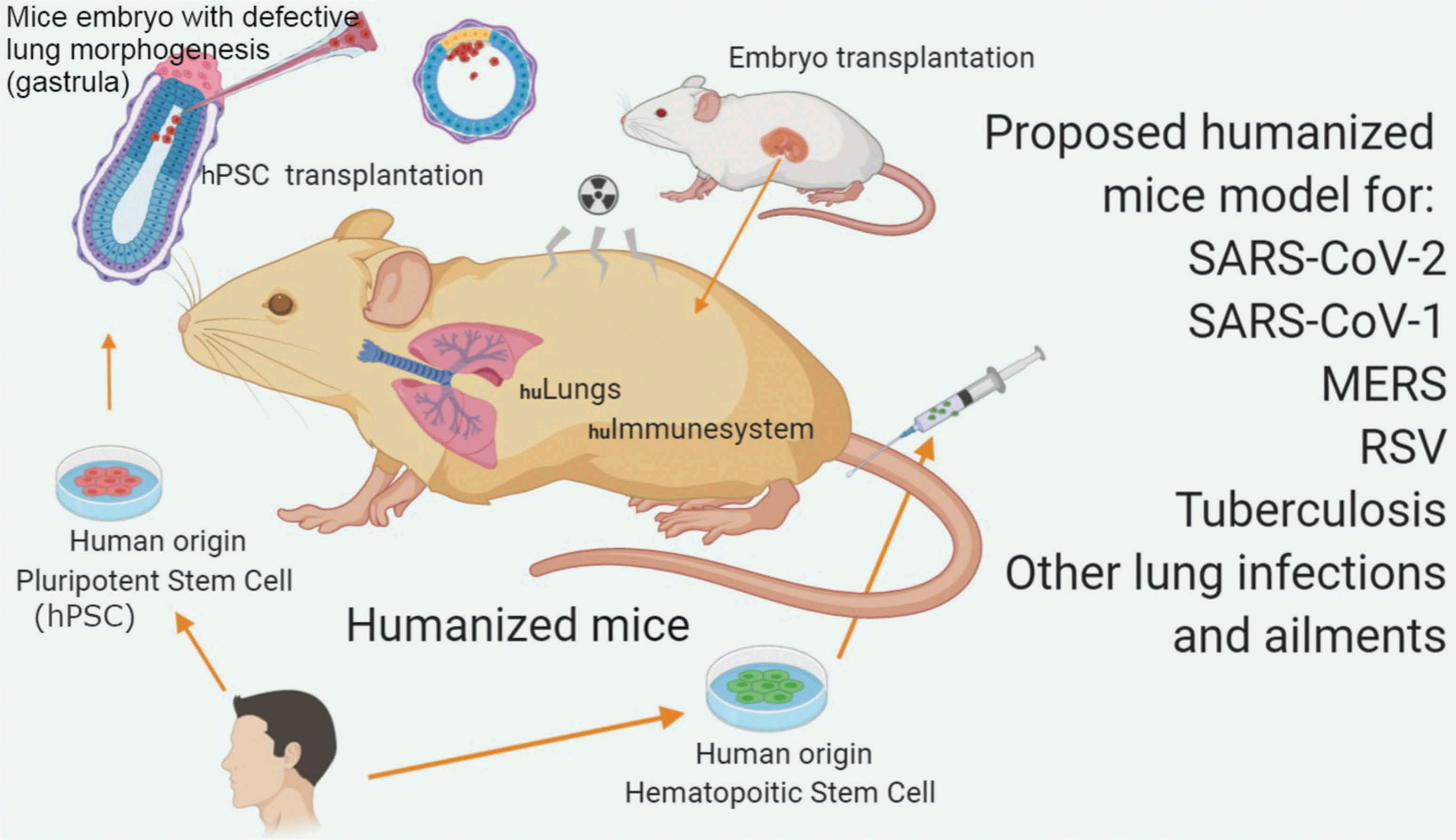
Proposed humanized mice model for respiratory diseases (based on the work of Wahl et al. and Mori et al.).

We lack a suitable animal model that can provide information on how coronaviruses, including SARS-CoV-2, behave in humans because they either do not share relevant physiology, do not mount an appropriate immune response, or do not present relevant clinical symptoms. Transgenic mice that express a human version of the protein hACE2 (originally developed to study SARS), ferrets, and non-human primates (NHP) appear to be susceptible to SARS-CoV-2 [2–5]; however, there are several problems associated with each of these animal models. hACE2 mice yield severe lung and brain infections with 100% mortality by day six post-infection with SARS-CoV virus but show very mild lung pathology and clinical phenotypes upon infection [3,6]. Ferrets, which have similar lung physiology to humans, show low pathogenicity for coronaviruses. NHP models have the advantage of immunological relatedness to the human immune system, but they are difficult and expensive to work with and present inconsistent clinical disease.

In light of recent events, two groundbreaking research studies have been published that could address some issues with recapitulating the normal infection cycle and pathogenesis of SARS-CoV-2. The first study focuses on a generation of humanized mice with ectopically transplanted human lung tissue and human immune cells via bone marrow transplantation, while the second study focuses on development of the mice with functional lungs by conditional blastocyst complementation using pluripotent stem cells, a step closer to having a mouse with humanized lungs [7,8].

Respiratory pathogens, including the newly emerged SARS-CoV-2, are classified among the major infectious disease problems and have been listed as one of the top 10 common causes of overall deaths globally. Building upon previous success in grafting human tissues in mice, Wahl et al. successfully developed human lung-only (LoM) mice by implanting human lung tissue in an immunocompromised mouse [7]. To recapitulate the human immune system, they also developed bone marrow–liver–thymic (BLT) mice model by transplanting human hematopoietic stem cells into the bone marrow of NSG mice strain, a non-obese diabetic–severe combined immunodeficiency (NOD-SCID) strain with a Prkdc mutation and an IL2Rγ deficiency that lacks mouse-specific T cells, B cells, and innate lymphoid cells that includes natural killer cells. Through these trials, Wahl et al. successfully demonstrated the infection of respiratory (MERS-CoV, RSV) and non-respiratory (HCMV, ZIKA, and Mycobacterium bovis BCG) human pathogens in their newly developed mice models.

In context to COVID-2019, an interesting feature to note is that coronavirus-induced immunity is short-lived and may last from 3 months to 1 year depending upon the viral strains. We still do not understand the molecular mechanism behind this weak immunity. A small animal model, such as BLT developed by Wahl et al., used in conjunction with a human hematopoietic cell could be useful in understanding immune responses such as B cell maturation and memory T cell establishment for coronaviruses. Increased knowledge about these processes is crucial for generating a robust vaccine candidate for COVID-19 and other lung-related infectious diseases.

In another study that leverages current understanding of the molecular organogenesis within lungs, Mori et al. employed a conditional blastocyst complementation approach to transplant a humanized lung and trachea in mice and other animal models [8]. They transplanted the pluripotent stem cells (PSCs) into the blastocyst of the severely genetically defective mutant mice. In the defective recipient mice embryos, PSCs were observed to take over the lung development process which would have otherwise resulted in fetal death due to defective lung agenesis. The team used Ctnnb1cnull and Fgfr2cnull mutant mice where the former gene specifies, and the later gene expands, the early respiratory endodermal progenitor cells. Lung organogenesis initiates when trachea and lung progenitor are collectively specified in the foregut endoderm by Wnt-β-catenin (Ctnnb1) activation. β-catenin is either at or near the top of the genetic hierarchy that executes the lung development program. It promotes respiratory progenitor characteristics both during lung initiation in the fetal lung and in stem cell maintenance in the adult lung. It is important to note that this pathway is a major regulator but not the sole player. Fibroblast growth factor receptor 2 (Fgfr2), the activation failure of which results not only in pulmonary agenesis but also impaired trophoblast formation and multiple defects such as limb agenesis during the later stages of development. To address this issue, Mori et al. applied a targeted gene-deletion strategy to conditionally delete Fgfr2 in the foregut endoderm immediately before the onset of lung organogenesis, which selectively affects the lung formation without affecting the limb formation. Although the PSCs (expressing GFP driven by lung-specific NKX2-1 promoter) complemented the defective Fgfr2 and formed the lobation or branching morphogenesis, it was unable to form the distal saccules for gas exchange and the pups died at birth. Mori et al. addressed this issue by using an a2i/VPA/LIF–treated PSC expressing GFP under CAG promoter and expressing high stage-specific embryonic antigen 1 in order to maintain the PSC’s pluripotency. a2i/VPA/LIF is a cocktail of stem cell culture medium which meditates self-renewal and enhanced expression of pluripotency-associated proteins (Oct4, Pecam, and Ssea1), epigenetic changes resulting in a more accessible (open) chromatin, and improved chimera formation efficiency [8].

Since respiratory infectious diseases are continuously on the rise, we believe that it is a good time to revisit the ethical considerations of humanizing mice with human organs – especially a mouse with human-like lungs (Figure 1). There have been significant advancements made in the stem cell technology and genetic engineering sector that could be utilized for faithful delivery of the human PSCs into the later stages of the mice embryonic development. Mori et ﻿al. ﻿have demonstrated such a proof of concept by utilizing the conditional blastocyst complementation approach for generating a functional lung in mice.
